# Development of Fast Analytical Method for the Detection and Quantification of Honey Adulteration Using Vibrational Spectroscopy and Chemometrics Tools

**DOI:** 10.1155/2020/8816249

**Published:** 2020-12-23

**Authors:** Omar Elhamdaoui, Aimen El Orche, Amine Cheikh, Brahim Mojemmi, Rachid Nejjari, Mustapha Bouatia

**Affiliations:** ^1^Laboratory of Analytical Chemistry, Team of Formulation and Quality Control of Health Products, Faculty of Medicine and Pharmacy, Mohammed V University in Rabat, Rabat, Morocco; ^2^Laboratory of Chemical Processes and Applied Materials, Faculty of Science and Technology, Sultan Moulay Slimane University, Beni-Mellal, Morocco; ^3^Faculty of Pharmacy, Abulcasis University, Rabat, Morocco; ^4^Laboratory of Pharmacognosy, Team of Formulation and Quality Control of Health Products, Faculty of Medicine and Pharmacy, Mohammed V University in Rabat, Rabat, Morocco

## Abstract

In this study, the Fourier transform mid-infrared (FT-MIR) spectroscopy technique combined with chemometrics methods was used to monitor adulteration of honey with sugar syrup. Spectral data were recorded from a wavenumber region of 4000–600 cm^−1^, with a spectral resolution of 4 cm^−1^. Principal component analysis (PCA) and hierarchical cluster analysis (HCA) were used for qualitative analysis to discriminate between adulterated and nonadulterated honey. For quantitative analysis, we used partial least-squares regression (PLS-R) and the support vector machine (SVM) to develop optimal calibration models. The use of PCA shows that the first two principal components account for 96% of the total variability. PCA and HCA allow classifying the dataset into two groups: adulterated and unadulterated honey. The use of the PLS-R and SVM-R calibration models for the quantification of adulteration shows high-performance capabilities represented by a high value of correlation coefficients *R*^2^ greater than 98% and 95% with lower values of root mean square error (RMSE) less than 1.12 and 1.85 using PLS-R and SVM-R, respectively. Our results indicate that FT-MIR spectroscopy combined with chemometrics techniques can be used successfully as a simple, rapid, and nondestructive method for the quantification and discrimination of adulterated honey.

## 1. Introduction

Adulteration of food for economic purposes is becoming a major problem in many countries due to the global trade growth. Currently, honey is considered one of the most susceptible foodstuffs to be targeted for adulteration. According to the standards of the *Codex Alimentarius*, honey is considered as the natural sweet substance obtained from the nectar or secretions of the living parts of plants [1]. In addition, honey's chemical composition and properties can be influenced by different factors such as the season, environmental conditions, and the botanical origin of the nectar source. The main chemical constituents of honey are sugar and water which are usually 80% carbohydrates (mainly fructose and glucose) and 17% water although other substances such as vitamins, proteins, enzymes, organic acids, biological compounds, and trace elements are also present in smaller amounts [2]. Thanks to its nutritional value, honey has been used in traditional medicine for millennia due to its potential antibacterial, anti-inflammatory, and antioxidant properties [4].

According to current regulations, honey is considered as a purely natural food that is never modified by additives, colorants, conservatives, or other substances [1]. The medicinal benefits and agreeable sweet taste stemming mainly from the natural composition of honey can explain the high cost of this product, which makes it susceptible to adulteration after olive oil [6] and milk [7]. The most common method of honey adulteration involves the addition of sugar syrup due to its similar composition to honey and its low price [8]. The increasing demand for honey in the market has made it absolutely necessary to establish reliable methods of analysis in order to ensure the authenticity of honey and to protect the consumer against any form of falsification.

Consequently, a lot of research has been devoted to address this authentication issue, in order to develop robust and efficient analytical methods suitable to provide information on the quality and the safety of honey. Such analytical methods can be divided into two general classes, those based on the analysis of chemical compounds of honey, such as high-performance liquid chromatography [10], gas chromatography [11], and C-isotope approach, and based on spectroscopic techniques, such as nuclear magnetic resonance [12], visible near-infrared [13], near-infrared (NIR) [14], and fluorescence [15].

Techniques based on the analysis of the molecular composition of honey (HPLC and GC), as reference methods, are typically time-consuming, require the use of expensive and environmentally polluting reagents and can only be carried out by qualified technicians.

Therefore, spectroscopic techniques such as visible spectroscopy (Vis), near-infrared spectroscopy (NIRS), mid-infrared spectroscopy (MIR), and fluorescence, combined with appropriate chemometrics multivariate methods, have gained importance in food safety control [16]. These methods have been recognized as a rapid and nondestructive alternative to detect and quantify the presence of adulterants since it provides important information on the presence of certain functional groups. It is also considered as a “fingerprinting technique,” which means that no two kinds of honey have the same FTIR spectra, whether in the number of peaks or in the intensity of the peaks [13].

The objective of this study is to develop a rapid method for the detection and quantification of honey adulteration using FT-MIR spectroscopy in combination with supervised and unsupervised chemometrics tools and to compare the predictive capability of the two regression methods PLS and SVM.

## 2. Materials and Methods

### 2.1. Sample Preparation

The pure honey samples from different nectar sources were directly obtained from the hives from different artisanal beekeepers located in the Rabat region. The samples were stored in plastic bottles at room temperature before analysis. A total of 8 honey samples were used for this study.

The series of artificial fraud honey samples were prepared by mixing one authentic honey with the range between 0.97% and 27% (w/w) of sugar syrup. Honey samples free of adulteration (0%) and pure sugar syrup samples were also prepared (100%):

(1) $$\begin{array}{c} \%\text{ adulteration}=\frac{\left(\text{mass of adulterant in sample}\right)*100}{\left(\text{total mass of sample}\right)}. \end{array}$$

After adding the sugar syrup, all samples were incubated in a water bath at about 35°C for 20 min until all the sugar crystals were melted, to get an adequate viscosity and mixed on a vortex to make sure homogeneity before analysis.

The samples were kept at room temperature to bring back the temperature of the sample to ambient, before FTIR analyses.

### 2.2. Spectrum Acquisition

Spectra were recorded on a single reflection diamond ATR (JASCO FTIR 460 PLUS (PIKE Technologies, Madison, USA)). The honey sample was placed directly on the ATR cell without any preparation. A drop of 20 *µ*L is sufficient to get great spectra on the basis of the selection of optimal signal to noise ratios. The spectra were recorded from 4000 to 600 cm^−1^, with a spectral resolution of 4 cm^−1^. Each spectrum was gathered and ratioed against the background spectrum of the clean crystal surface in order to present the spectra in absorbance. Three replicas of every sample were recorded at room temperature. After each measurement, the crystalline surface was washed with isopropanol solution and dried with a soft paper.

### 2.3. Data Analysis

In this study, various statistical methods have been used for processing and evaluation of spectral data obtained by ATR FT-MIR spectroscopy. To ensure full representation and exploration of the dataset, we began analyzing the results by PCA and HCA.

Principal component analysis (PCA) is an unsupervised model recognition that generally represents the first step in exploratory data analysis to identify groups in the collected data. PCA is very useful when there is a large amount of quantitative data to be processed and interpreted. Its purpose is to extract the most important information from the data table and express it by projecting the data on a set of new orthogonal variables called principal components (PCs). The PCs describe, in descending order, the largest variations between characteristics, and because they are calculated to be orthogonal to each other, each PC can be interpreted independently. This gives an overview of the data structure by revealing the relationships between objects and the detection of deviant features [17, 18].

HCA is a clustering technique that explores the arrangement of samples in groups and in the midst of groups portraying a hierarchy. The result of HCA is often presented in a dendrogram, a plot that illustrates the arrangement of samples and its correlation in tree form. The distance between two samples in the dendrograms is measured to determine the similarity between these samples based on their different attributes [19].

Support vector machine (SVM) method is part of the supervised learning algorithms. It solves the problem of pattern recognition. It consists of finding an optimal separator that maximizes the margin between two classes of data, using a limited set of learning sequences [20].

PLS-R is a regression technique used mainly for multivariate data including spectral data. It is commonly applied for the prediction and quantification of certain chemical parameters in agrifood or pharmaceutical products [21].

SVM-R is a regression method that is part of the learning machine method that allows to estimate the functional dependence between the dependent variable (*y*) which is the response and a group of independent variables (*x*) [22].

The calibration approach for quantification of the adulteration was deployed in two steps, calibration and validation [23]. The performance of the model is examined by the root mean square error of calibration (RMSEC), root mean square error of cross-validation (RMSECV), and regression coefficient (*R*^2^). The selected model is then utilized to determine the concentration of samples from an independent (or external) set of predictions. The predictive strength of the model is evaluated from the root mean square of prediction (RMSEP) [24, 25]. The lower RMSE and the higher *R*^2^ indicate a good quality of the prediction.

### 2.4. Chemometrics Software

Partial least-squares (PLS) and support vector machine (SVM) techniques were also performed using Unscrambler software, Version 10.4., which were used for quantitative analysis. Discriminant analysis, PCA, and HCA were utilized to detect the presence of adulterants and to separate between the authentic and adulterated honey.

## 3. Results and Discussion

### 3.1. Classification of Pure and Adulterated Honey

To properly explore the spectral dataset, we applied some unsupervised chemometrics methods such as principal component analysis (PCA) and cluster analysis.

PCA was firstly applied based on spectral data, processed by the Savitzky–Golay algorithm and normalization to reduce undesired spectral effects. The spectra have been divided into two categories, that of adulterated honey with sucrose and that of pure honey, in order to represent all the data in a two-dimensional space.

**Figure 1:**
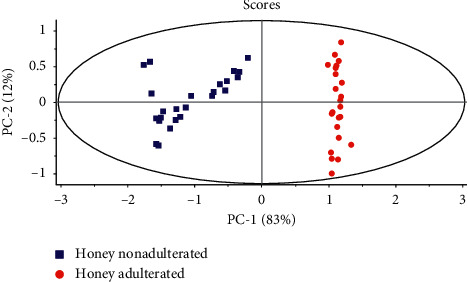
PCA scores plot of samples of pure honey and adulterated honey.

The application of PCA shows that the first two components represent 95% of the total variability of the data. The score plot shows that there is strong discrimination between the two groups of honey, this discrimination is ensured essentially by the first main component, the graph also makes it possible to show that there is an intragroup dispersion in the groups of honey (Figure 1), and this dispersion is essentially due to the different botanical origin of honey used [26, 27].

HCA aims to construct a group division, where the variables are joined together hierarchically from the nearest one (which resemble the most) to the farthest and then expressed in a dendrogram or tree [28].

**Figure 2:**
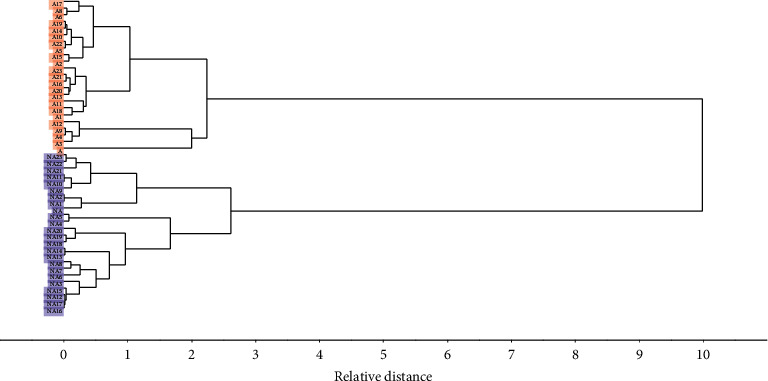
Dendrogram from an HCA with single linkage of adulterated and nonadulterated honey samples using the squared Euclidean distance.

Figure 2 illustrates the dendrogram for the classification study of samples based on the HCA technique. It was possible to separate two groups, adulterated and nonadulterated honey. The technique was successful considering the data centered on the mean, and the single linkage algorithm is used to define the proximity between samples [29]. The similarity of the samples of honey is found considering the distance between them. It was observed that the types of honey samples are successfully differed from each other. Spectral-based HCA assumes that samples with similar spectral profiles are chemically related and should be assigned to a single group [30].

**Figure 3:**
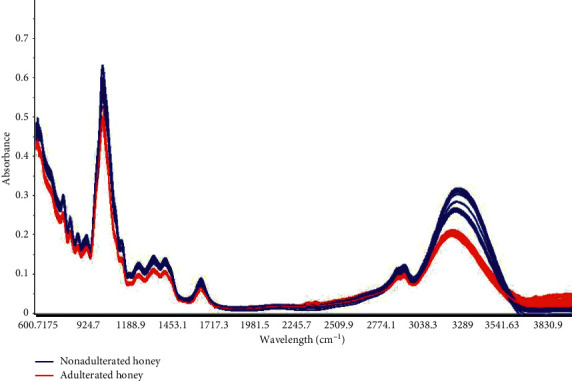
Mid-infrared spectrum of pure honey and adulterated honey.

With the application of HCA and PCA techniques in the MIR spectra of honey, it was possible to separate and differentiate pure honey from adulterated honey, enabling clear discrimination between the two groups of honey according to their purity. This strong discrimination is explained by the spectral difference between adulterated and nonadulterated honey, as shown in Figure 3, where it is observed that there is a difference in the spectral intensity of the bands between 3090 cm^−1^ and 3513 cm^−1^ and also between 600 cm^−1^ and 1456 cm^−1^, which can be explained by the difference in the composition of the samples [31].

To develop a rapid method, able to authenticate honey adulteration, based on mid-infrared spectroscopy, a classification model SVM (type C-SVC) was developed by using a linear kernel algorithm. Cross-validation has been performed on the training data to select various parameters of the model among a set of given values. This validation option solves the problem of overfitting by limiting the complexity of the model or by providing independent control of the model performance.

**Figure 4:**
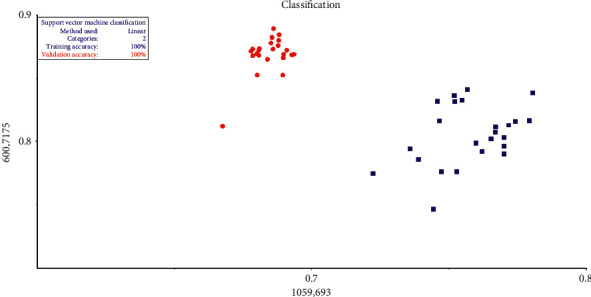
Classification by support vector machine (SVM) using linear kernel algorithm.

The use of the SVM classification method shows a strong capacity to differentiate between pure honey and adulterated honey, and this capacity is expressed by the accuracy of discrimination which represents 100%, as shown in Figure 4. The method of cross-validation applied during the validation of all the tests shows that the proposed model appears to be an efficient chemometrics approach.

The use of this method also shows that as a PCA, there is high intragroup variability in pure honey; therefore, this tool is also capable of providing information not only on adulteration but also on the botanical origin of honey.

From these results, it can therefore be concluded that mid-infrared spectroscopy combined with PCA, HCA, and SVM chemometrics learning tools has a very high ability to discriminate between adulterated and nonadulterated honey during a very short period of time and with an accuracy of 100%.

### 3.2. Quantification of Honey Adulteration

If the identity of the adulterant is known, it is possible to quantify the amount of adulterant present. This is the preparation and measurement of the IR spectra of standard honey mixtures with adulterant.

PLS-R and SVM-R calibration models were performed on 56 adulterated samples ranging from 0.93% to 27.27% to determine the relationship between predictor variables (absorbance) and the physicochemical characteristics of honey and, more precisely, to construct chemometrics models capable of predicting and quantifying the adulteration of honey by sugar syrup.

The PLS and SVM regression models were performed on a spectral region of medium infrared spectroscopy from 4000 cm^−1^ to 600 cm^−1^ without and with spectral preprocessing.

The prediction quality of the constructed models was examined using the root mean square error of calibration (RMSEC), the coefficient of regression *R*^2^, and the root mean square error of cross-validation (RMSECV). To validate the developed PLS and SVM models, the leave-one-out (LOO) cross-validation method was used. In this technique, one sample at a time was excluded, and then, the sample removed was predicted with a model constructed by the remaining samples. This procedure was repeated until each sample was excluded once. The predictive quality of the multivariate regression models developed should be evaluated on samples other than those used for the construction of the PLS and SVM models using external validation.

The parameters of the PLS and SVM regression, with and without spectral correction, are listed in Table 1. This table also summarizes the performance and calibration parameters in terms of the multiple determination coefficient (*R*^2^), the root mean square error of calibration (RMSEC), and the root mean square error of cross-validation (RMSECV). The high value of *R*^2^ and the low value of RMSEC and RMSECV indicate the good performance and accuracy of the PLS- and SVM-constructed models.

**Figure 5:**
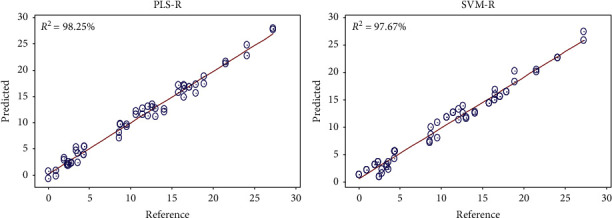
Example of PLS-R and SVM-R calibration models of honey adulteration.

The PLS and SVM regression models show that there is a high correlation between the MIR-TF spectra of honey and their adulteration rate by sugar syrup, as shown in Figure 5. Generally, several mathematical models have been constructed with different spectral preprocessing to develop high-performance models that have a good quality of prediction of the adulteration rate in honey. Table 1 shows that these developed PLS and SVM regression models have very high performance with a correlation coefficient between 96.34% and 98.86% and an error between 0.96 and 1.41 for calibration results. And for cross-validation results, the correlation coefficient is between 85.26% and 95.12%, and the error is between 1.68 and 2.84.

From these results, we also note that the calibration models developed by the PLS regression have better regression parameters than those developed by the SVM regression. As can be seen in Table 1, the PLS regression has a correlation coefficient greater than 98% and an error of less than 1 for the calibration results and a correlation coefficient greater than 92% with a minimum error of 1.68 for the cross-validation results. These results are considered more reliable compared to those obtained by the SVM regression.

**Figure 6:**
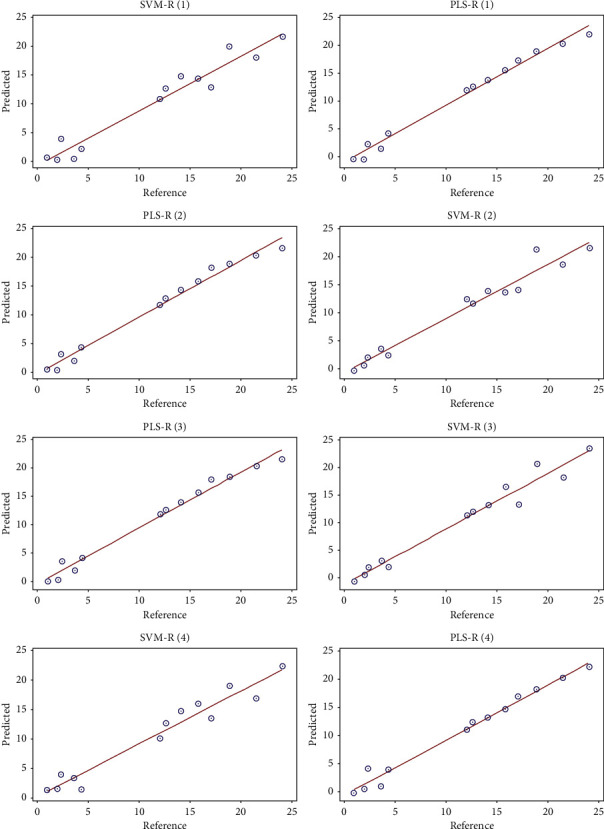
Results of the external validation by PLS-R and SVM-R of the different models built, representing the predicted values according to the reference values.

To verify the capacity of these models in the quantification of honey adulteration, 13 samples were used for external validation of these models, the test sample was analyzed, and the results of the test group data were predicted using the PLS-R and SVM-R models. The results for predicted and actual percentages are mentioned in Table 2, and Figure 6 clearly shows the concordance between real and predicted percentages of adulteration. Prediction quality *R*^2^ values are greater than 98% for PLS regression and 95% for SVM regression with a mean square error of less than 1.12 for PLS-R and 1.85 for SVM regression. The predicted values are very consistent with real values. The results indicate that the method proposed in this work is feasible for the detection and quantification of adulteration of pure honey with sugar syrup. As previously mentioned in the calibration and cross-validation results, the PLS-R regression shows high performance compared to the SVM regression.

The quantification of sugar in honey was studied by Rios et al. [32]. The lowest value of RMSEC was 0.377%; however, the model showed a high value of RMSEP of 3.150%, and *R*^2^ was >0.999 for the calibration dataset and also >0.999 for the validation dataset.

Sivakesava and Irudayaraj [33] developed PLS models capable of detecting a low sugar concentration in honey (0.5%) with RMSEP between 2.8 and 3.6%, values of the same order of amplitude as in this work.

## 4. Conclusion

The present work has been designed to evaluate the capacity of MIR spectroscopy coupled with chemometrics algorithms to detect and quantify the adulteration of honey by sugar syrup; for this purpose, multivariate analysis and classification methods such as PCA, HCA, and SVM have been used to discriminate between adulterated honey and pure honey, and their application leads to a perfect classification of both groups of honey. This classification capacity is represented by the percentage of discrimination accuracy that reaches 100% using the SVM classification method.

Besides, the application of PLS and SVM regression methods has shown a very high capacity in quantifying the percentage of sugar syrup adulteration, which is expressed by the high value of the correlation coefficient and the low value of the prediction error using cross-validation and external validation. The capability of the proposed method to quantify low adulteration has been successfully demonstrated.

These results show that the application of this technique can be a promising tool to control the quality of honey because it is fast, nondestructive, and simple to use. It would also be interesting to study more adulterants and/or other high-quality honey samples to enhance the field of application of this technique.

## Figures and Tables

**Table 1:** Performance parameters of PLS-R and SVM-R.

Regression	Preprocessing	Calibration		Cross-validation	
		*R* ^2^(%)	RMSEC	*R* ^2^(%)	RMSEP
PLS-R (1)	Without preprocessing	98.25	0.98	93.91	1.91
SVM-R (1)		97.67	1.19	85.26	2.84

PLS-R (2)	Detrend polynomial degree 1	98.17	1.00	94.35	1.79
SVM-R (2)		97.96	1.10	93.50	1.92

PLS-R (3)	Detrend polynomial degree 2	98.30	0.96	94.93	1.68
SVM-R (3)		97.96	1.07	92.34	2.06

PLS-R (4)	Detrend polynomial degree 3	98.86	0.79	95.12	1.71
SVM-R (4)		97.57	1.11	90.43	2.30

**Table 2:** Performance of the PLS-R and SVM-R models by external validation using FTIR.

External validation			
Regression	Preprocessing	*R* ^2^	RMSEP
PLS-R (1)	Without preprocessing	98.7	1.11
SVM-R (1)		94.9	2.08

PLS-R (2)	Detrend polynomial degree 1	98.3	1.05
SVM-R (2)		96.5	1.71

PLS-R (3)	Detrend polynomial degree 2	98.3	1.09
SVM-R (3)		96.6	1.70

PLS-R (4)	Detrend polynomial degree 3	98.3	1.25
SVM-R (4)		95.0	1.92

## Data Availability

The data used to support the findings of this study are available on request from the corresponding author.
